# Evaluation of peak cough flow in Brazilian healthy adults

**DOI:** 10.1186/1755-7682-5-25

**Published:** 2012-09-28

**Authors:** Fernanda EF Cardoso, Luiz Carlos de Abreu, Rodrigo Daminello Raimundo, Natália ANM Faustino, Suellen F Araújo, Vitor E Valenti, Monica Akemi Sato, Silvia RG Martins, Jamili A Torquato

**Affiliations:** 1 Universidade Cruzeiro do Sul. Av. Regente Feijó 1295, 03342-000, São Paulo, SP, Brasil; 2 Laboratório de Delineamento de Estudos e Escrita Científica. Departamento de Morfologia e Fisiologia, Faculdade de Medicina do ABC. Av. Príncipe de Gales, 821. Príncipe de Gales, 09060-650, Santo André, SP, Brasil; 3 Departamento de Fonoaudiologia, Faculdade de Filosofia e Ciências, Universidade Estadual Paulista, UNESP. Av. Hygino Muzzi Filho 737, 17525-000, Marília, SP, Brasil

**Keywords:** Respiration, Cough, Peak expiratory flow rate, Physiology

## Abstract

**Introduction:**

In this study we aimed to evaluate the peak cough flow (PCF) in healthy Brazilian subjects.

**Methods:**

We evaluated 484 healthy subjects between 18 and 40 years old. Subjects were seated and oriented were asked to perform a maximal inspiration followed by a quick, short and explosive expiration on the peak flow meter. Three measures were carried out and recorded the average of the three results for each individual.

**Results:**

The PCF values ranged between 240 and 500 L/min. The PCF values were lower in females than in males. The PCF was inversely proportional to age.

**Conclusion:**

The values for Brazilian adult healthy subjects regarding PCF were between 240 and 500 L/min.

## Introduction

Among the methods used to evaluate pulmonary function, we may include some parameters such as forced vital capacity [1], total lung capacity [2] and peak expiratory flow (PEF) [3]. PEF is a maximum flow generated during a forced expiration after lung inflation, i.e., starting from total lung capacity (TLC) [3-5].

Respiratory disorders impairs quality of life in several ages [6-8]. Along with PEF, peak cough flow (PCF) has provided clinical data to identify common variations in the respiratory muscles function [3-5]. Respiratory and neuromuscular diseases induce respiratory muscles weakness, resulting in accumulation of secretions in the airways, leading to pneumonia development, tracheal intubation and tracheostomy [9].

Due to respiratory changes, which results in ineffective cough, it is believed that the development of a PCF normative values in normal individuals from Brazil is important to early detect values below the reference from normal and intervene in respiratory muscle training and cough. Therefore, we aimed to evaluate the PCF in Brazilian healthy adult subjects.

## Methods

We selected 484 healthy Caucasian Brazilian individuals according to spirometric values from public institutions in São Paulo city between March 2009 and August 2009, which were submitted to the PCF test. All subjects presented normal respiratory function according to the American Thoracic Society [10]. We considered the following exclusion criteria: smokers, cardiopulmonary disorders, neurological and other impairments that prevent the subject known to perform the procedures, treatment with drugs that influence respiratory function and body mass index (BMI) below 20 kg/m^2^ and above 25 kg/m^2^. Participants in this study signed a consent form. The study was approved by the Ethics in Research of the School of Medicine of ABC (No. 009/2009).

Among the subjects evaluated, 232 (47.9%) were males and 252 (52.1%) were females, they aged between 18 and 40 years old. We included 230 men (50%) and 230 women (50%) for the preparation of the values, distributed according to age as shown in Table 1. The exclusion of 24 individuals was based on inadequate comprehension of the PCF test, installation of lung disease, smokers, values not in accordance with the American Thoracic Society [10] and recent surgeries.

**Table 1:** **Average peak cough flow values (L/min) in men and women according to age**

**Age (years)**	**Men - Mean ±** **SD (n)**	**Women - Mean ±** **SD (n)**
**18**	499 ± 51 (10)	355 ± 31 (10)
**19**	486 ± 48 (10)	320 ± 29 (10)
**20**	484 ± 47 (10)	327 ± 34 (10)
**21**	435 ± 49 (10)	314 ± 28 (10)
**22**	428 ± 36 (10)	313 ± 25 (10)
**23**	407 ± 39 (10)	313 ± 34 (10)
**24**	406 ± 43 (10)	302 ± 33 (10)
**25**	494 ± 34 (10)	302 ± 31 (10)
**26**	486 ± 37 (10)	310 ± 30 (10)
**27**	484 ± 45 (10)	304 ± 24 (10)
**28**	439 ± 35 (10)	298 ± 23 (10)
**29**	430 ± 41 (10)	297 ± 31 (10)
**30**	426 ± 44 (10)	296 ± 38 (10)
**31**	410 ± 39 (10)	286 ± 25 (10)
**32**	399 ± 32 (10)	295 ± 36 (10)
**33**	380 ± 41 (10)	285 ± 21 (10)
**34**	380 ± 33 (10)	282 ± 29 (10)
**35**	376 ± 32 (10)	280 ± 34 (10)
**36**	381 ± 43 (10)	278 ± 35 (10)
**37**	360 ± 33 (10)	279 ± 21 (10)
**38**	347 ± 49 (10)	262 ± 30 (10)
**39**	337 ± 39 (10)	258 ± 23 (10)
**40**	316 ± 38 (10)	242 ± 34 (10)

N=number of people; l/min=litters per minute; SD=standard deviation.

Subjects were seated and asked to perform a voluntary couch. The volunteers performed a maximal inspiration, followed by a quick, short and explosive expiration on the peak flow meter. The difference from the peak expiratory flow is the higher glottis pressure and the higher resistance induced by the closed glottis, which characterizes a forced cough. Three measures were carried out and we considered the average of three results for each individual. It was performed a total of 1380 PCF measures in 460 subjects (230 men).

The peak flow meter used was the Mini-Wright Peak Flow Meter Clement Clarke International Ltd., portable, easy to handle and made of plastic material. It was used in order to assess the strength and speed exerted by the expiration in liters per minute (L/min). During the period we used our mechanical tool we did not realize any damage of the instrument [5,11].

We applied the Kolmorogov-Smirnov normality test in order to evaluate the distributions. Considering that all distributions were parametric, we applied the Student t test to compare PFC between men and women and the Pearson correlation coefficient to verify the correlation between age and PFC. Differences were considered significant when the probability of a type I error was less than 5% (p < 0.05). We used the software SPSS 11.5 for Windows.

## Results

The PCF values ranged from 240 to 500 L/min in relation to the whole group. In males mean PCF values ranged from 316 L/min to 499 L/min and in females it ranged from 242 L/min to 355 L/min.

**Figure 1:**
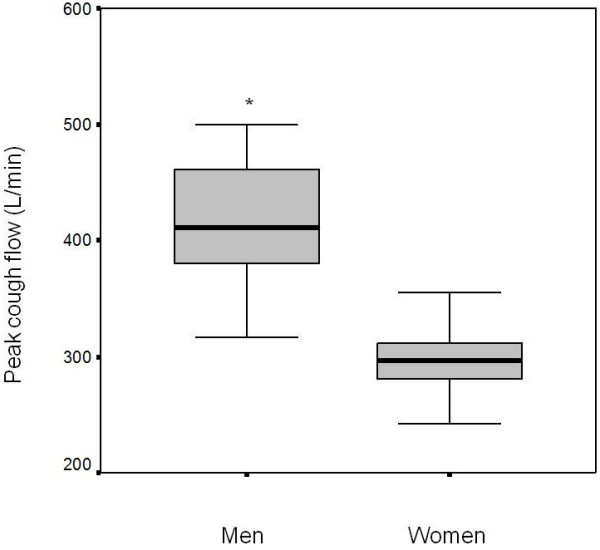
Maximal peak cough flow according to gender. *p < 0.05: Different from women.

Figure 1 displays data regarding comparison of PCF between males and females. We noted that males presented higher values than females.

In Table 1 we observed that there was a significant decrease in the PCF in men according to age. In women we found that there was a decrease in PCF without major modifications. The female data are homogeneous compared to males, which presented a major difference between ages.

The both groups presented a relative decrease of the PEF according to the age. There was a significant correlation between PEF and age for both groups (r = −0.851; p = 0.001 for men and r = −0.941; p = 0.001 for women).

## Discussion

Based on our data, the values for PCF in Brazilian healthy adults ranged between 240 and 500 L/min. The PCF correlates with respiratory muscle strength, especially with inspiratory muscle strength. In the inspiratory phase of cough, inhalation of high volumes promotes a relationship of time and tension of the muscles and increases the elastic recoil of the respiratory system, optimizing expiratory pressures. The muscle strength to inhale appropriate volumes lead to chest expansion and the muscle force to increase intrathoracic pressure are important to generate effective cough and high flows [12].

One of the limitations of the voluntary evaluation of PCF is the understanding and cooperation of the subjects. Also, the cough reflex mechanism would be triggered by a reasonable way to replacing the method of evaluation. The differences in voluntary and reflex cough go beyond the way they are triggered. However, there are differences in how muscle action is triggered [13].

Our data showed that there are minimal changes between ages, and we observed in our sample that it may not be changed due to the no comprehension of the subject, since we excluded those who did not comprehend the test. During the voluntary cough the expiratory and accessories muscles action only generates one PCF. On the other hand, during the reflex cough the muscle action is harmonious and simultaneous, generating two or more peak expiratory flow of lower amplitude [13].

According to our results, the PCF values ranged from 240 to 500 L/min. According to the literature, in healthy individuals, the average PCF is higher than 300 L/min in Caucasian European subjects. Additionally, the PCF must be higher than 160 L/min for an effective cough according to the literature [14].

Our findings were obtained from healthy subjects without decrease in muscle strength. The consequence of an inspiratory muscle weakness is the loss of ability to perform a deep inspiration, which is necessary to maintain peripheral alveolar ventilation in addition to affecting the first phase of cough (deep breath). The weakness of the expiratory muscles may be seen in a reduction in maximal expiratory pressure, reduced ability to perform a deep expiration and cough [15].

We found some difficulties such as lack of reference to studies in relation to similar themes or methodologies on the PCF values. Furthermore, there were difficulties in the selection of the subjects, which limited the study sample.

The intent of this study was to evaluate PCF in a cohort of healthy Brazilian subjects. However, age range was not large enough; therefore, it is hard to develop prediction equations from the values we obtained. We suggest future studies to provide prediction equations for this variable. This equation would provide a reference against which measurements from patients with respiratory muscle weakness could be compared in order to identify early those at risk of problems with cough and secretion clearance. While normative values for other aspects of respiratory function such as PEF, TLC and maximum inspiratory and expiratory muscle strength are available, this is not the case for PCF, and therefore, normative data would be very important. This information could be used in developing prevention programs by identifying and intervening in patients at risk.

## Conclusion

In this study we investigated PCF in Brazilian healthy adults (between 240 and 500 L/min). The PCF was inversely proportional to age, the higher the age the lower the PCF in both genders.

## Competing interest

The authors declare that they have no competing interest.

## Authors’ contributions

FEFC, LCA, RDR, NANMF, SFA, VEV, MAS, SRGM, JAT participated in the acquisition of data and revision of the manuscript. All authors conceived of the study, determined the design, performed the statistical analysis, interpreted the data and drafted the manuscript. All authors read and gave final approval for the version submitted for publication.
